# Size dependence of the magnetic properties of Ni nanoparticles prepared by thermal decomposition method

**DOI:** 10.1186/1556-276X-8-446

**Published:** 2013-10-28

**Authors:** Xuemin He, Wei Zhong, Chak-Tong Au, Youwei Du

**Affiliations:** 1 National Laboratory of Solid State Microstructures and Jiangsu Provincial Laboratory for NanoTechnology, Department of Physics, Nanjing University, Nanjing 210093, China; 2 Department of Chemistry, Hong Kong Baptist University, Hong Kong 852, China

**Keywords:** Size dependence, Curie temperature, Cohesive energy, Magnetically inactive layer

## Abstract

By means of thermal decomposition, we prepared single-phase spherical Ni nanoparticles (23 to 114 nm in diameter) that are face-centered cubic in structure. The magnetic properties of the Ni nanoparticles were experimentally as well as theoretically investigated as a function of particle size. By means of thermogravimetric/differential thermal analysis, the Curie temperature *T*_C_ of the 23-, 45-, 80-, and 114-nm Ni particles was found to be 335°C, 346°C, 351°C, and 354°C, respectively. Based on the size-and-shape dependence model of cohesive energy, a theoretical model is proposed to explain the size dependence of *T*_C_. The measurement of magnetic hysteresis loop reveals that the saturation magnetization *M*_S_ and remanent magnetization increase and the coercivity decreases monotonously with increasing particle size, indicating a distinct size effect. By adopting a simplified theoretical model, we obtained *M*_S_ values that are in good agreement with the experimental ones. Furthermore, with increase of surface-to-volume ratio of Ni nanoparticles due to decrease of particle size, there is increase of the percentage of magnetically inactive layer.

## Background

The transition metal nickel shows distinct magnetic and catalytic properties [1,2]. In nanostructure, Ni has great application potential in fields such as pharmaceutical synthesis [3], magnetic biocatalysis [4], biomolecular separation [5], and biosensor [6]. In the literatures, there are reports on the preparation and properties of novel Ni nanomaterials such as sea urchin-like Ni nanoparticles [7], tetragonal Ni nanoparticles [8], hexagonal close-packed (*hcp*) Ni nanoparticles [9], conical Ni nanorods [10], triangular and hexagonal Ni nanosheets [11], and Ni nanochains [12]. It is known that the performance of technological devices is greatly influenced by the purity, structure, shape, and size of Ni nanoparticles. Hence, it is of great significance to prepare high-quality Ni nanomaterials of specificity using convenient and low-cost methods.

For the fabrication of Ni nanoparticles, methods such as sputtering [13,14], solution glow discharge [15], pulsed laser ablation [6], reversed micelles [16], thermal decomposition [17-20], and wet chemical reduction [7,21,22] are used. Among them, the ones based on thermal decomposition are preferred. The single-step process is facile, environment-benign, inexpensive, and reproducible, yielding high-quality Ni powders that can be controlled in terms of structure, morphology, size, and size distribution. It should be pointed out that pure Ni nanoparticles are difficult to prepare because they are easily oxidized. One of the ways to evade the formation of oxide or hydroxide is to carry out the pyrolysis process in organic media. For example, relatively large Ni nanoparticles were prepared through thermal decomposition of Ni(ac)_2_ 4H_2_O in oleylamine in the presence of 1-adamantane carboxylic acid (ACA) and trioctylphosphine oxide (TOPO) [19]. Furthermore, structure-controlled Ni nanoparticles were prepared via thermal decomposition of Ni(ac)_2_ 4H_2_O in long-chain amines that acted both as solvent and reducing agent [23]. More interestingly, trigonal Ni nanoparticles were prepared by reacting Ni(COD)_2_ in tetrahydrofuran with tetra-*n*-octylammonium carboxylates (as reductant and stabilizer) [24]. Despite the synthesis of superior Ni nanomaterials through the pyrolysis of organometallic salts in organic media has been known for quite some time, the synthesis of Ni nanoparticles 20 to 100 nm in size has only been reported lately.

Bulk Ni exhibits a rock-salt structure and is ferromagnetic (Curie temperature of bulk Ni *T*_Cb_ = 358°C) and electroconductive [25]. In contrast to the bulk counterparts, Ni nanoparticles show magnetic parameters (such as Curie temperature *T*_C_, saturation magnetization *M*_S_, and coercivity *H*_C_) that vary with particle size, usually in a non-linear fashion. Despite the endless number of reports on magnetic studies of Ni nanoparticles [1,10,18,26-30], the influence of particle size on the magnetic properties has not been systematically studied. It is envisaged that the application of Ni nanoparticles can be widened once the intrigue relationship between magnetic properties and particle size of Ni nanomaterials can be delineated.

Herein, we report a facile and reproducible process for large-scale synthesis of face-centered cubic (*fcc*) Ni nanoparticles (spherical and 23 to 114 nm in diameter). We controlled the size of Ni nanoparticles by regulating the synthesis temperature. We studied the influence of particle size on Curie temperature, saturation magnetization, and coercivity. We establish the size dependence of magnetic properties based on experimental as well as theoretical results, and comment on the critical size of Ni nanoparticles and percentage of magnetically inactive layer.

## Methods

Spherical Ni nanoparticles were prepared through high-temperature reductive decomposition of nickel(II) acetylacetonate ([Ni(acac)_2_]) with oleic acid (OA) and oleylamine (OAm) both as surfactant and solvent. In a typical synthesis process, [Ni(acac)_2_] (0.51 g, 2 mmol), OA (12 mL, 38 mmol), and OAm (18 mL, 55 mmol) were mixed and stirred in a three-necked flask. The mixture was heated at 130°C in H-320 conduction oil for 30 min under an argon atmosphere to give a clear emerald solution. Under the Ar blanket, the solution was heated to 240°C and kept at this temperature for 1 h. Then, the solution was cooled down to room temperature to obtain a black colloidal solution. A black precipitate was separated upon the addition of ethanol and hexane followed by centrifugation. The black substance was washed using a mixture of ethanol and toluene, and vacuum dried in an oven at 60°C overnight. Under similar reaction conditions, the size of the spherical Ni nanoparticles was tuned from 23 to 114 nm by simply increasing the reaction temperature from 240°C to 285°C as shown in Table 1.

**Table 1:** Size and magnetic parameters for the Ni-particle samples obtained at different temperatures

**Sample**	***T*** **(°C)**	***D*** **(nm)**	***T***_**C**_**(°C)**	***M***_**S**_**(emu/g)**	***M***_**r**_**(emu/g)**	***H***_**C**_**(Oe)**	***t*/*D*** **(%)**
a	240	23	335	40.47	6.81	156	4.26
b	255	45	346	44.33	9.67	119	3.09
c	270	80	351	46.47	12.56	76	2.43
d	285	114	354	47.80	17.59	17	2.03

X-ray diffraction (XRD) patterns of all the samples were obtained by using an X-ray diffractometer (Philips X’pert, Philips, Amsterdam, The Netherlands) with Cu K*α* radiation. To examine the morphology and particle sizes, a field-emission scanning electron microscope (SEM) (Hitachi S-4800, Hitachi Ltd., Chiyoda-ku, Japan) was used. High-resolution transmission electron microscope (TEM) image and selected-area electron diffraction (SAED) pattern were obtained from a FEI Tecnai G2-F30 instrument (FEI, Hillsboro, OR, USA) operated at accelerating voltage of 300 kV. In the TEM experiment, the incident electron beam was along the direction perpendicular to the sample. The sample was transferred onto a copper grid by solution dripping with the sample powder under sonication in ethanol. The Curie temperature was measured using a thermogravimetric (TG)/differential thermal analysis (DTA) instrument (Scinco STA-1500, Scinco Co. Ltd., Seoul, South Korea) equipped with a piece of Nd_2_Fe_14_B permanent magnet during sample heating (up to 500°C) in argon at a rate of 10°C/min. For comparing, the TG/DTA measurement of a bulk Ni sample (nickel sphere) was conducted under the same conditions. Magnetic measurements were carried out with a vibrating sample magnetometer (VSM) (Lake Shore 7300, Lake Shore Cryotronics, Inc., Westerville, OH, USA). The hysteresis was recorded for powder samples in gelatin capsule, and the hysteresis loops were obtained in a magnetic field up to ±10 kOe. Magnetization versus temperature (*M*-*T*) curves were measured in the range 30°C to 400°C using an applied magnetic field of 5 kOe.

## Results and discussion

**Figure 1:**
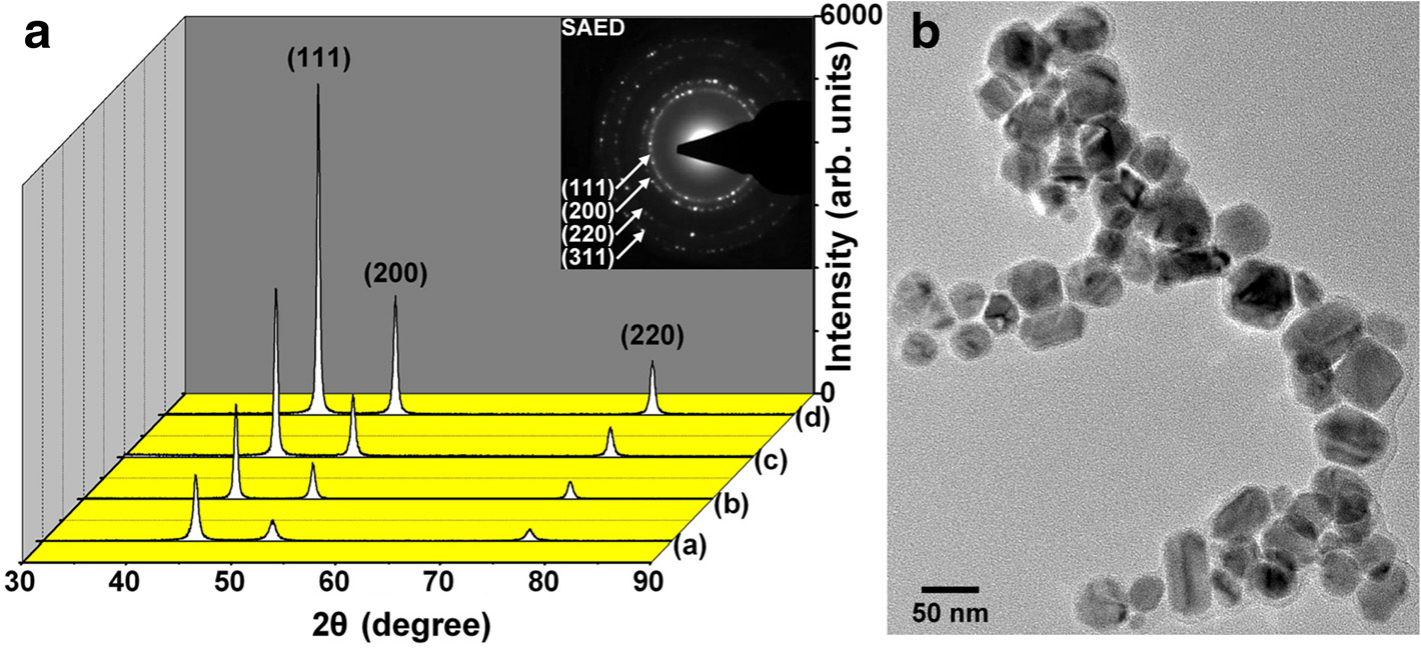
**XRD patterns (a) and HRTEM image (b).** Ni nanoparticles formed at (a) 240°C, (b) 255°C, (c) 270°C, and (d) 285°C. SAED pattern and HRTEM image relative to Ni nanoparticles formed at 255°C.

Figure 1a shows the powder XRD patterns of the Ninanoparticle samples synthesized at 240°C, 255°C, 270°C, and 285°C. All samples are single-phase with face-centered cubic (*fcc*) structure, and no phase of NiO or other impurity is observed. The three obvious peaks (2*θ* = 44.58°, 51.90°, 76.54°) can be assigned to the (111), (200), and (220) planes of Ni crystal lattice, respectively. It is clear that there is peak broadening upon decrease of reaction temperature. In other words, the particle size of the prepared Ni nanoparticles increases with increasing reaction temperature, and this is confirmed in SEM analysis (show later). As can be seen in the SAED pattern (inset of Figure 1a), perfect cubic symmetry can be clearly identified for the polycrystalline Ni nanoparticles. The high-resolution TEM image in Figure 1b presents the granular morphology and further reveals the surface nature of magnetically dead layer.

**Figure 2:**
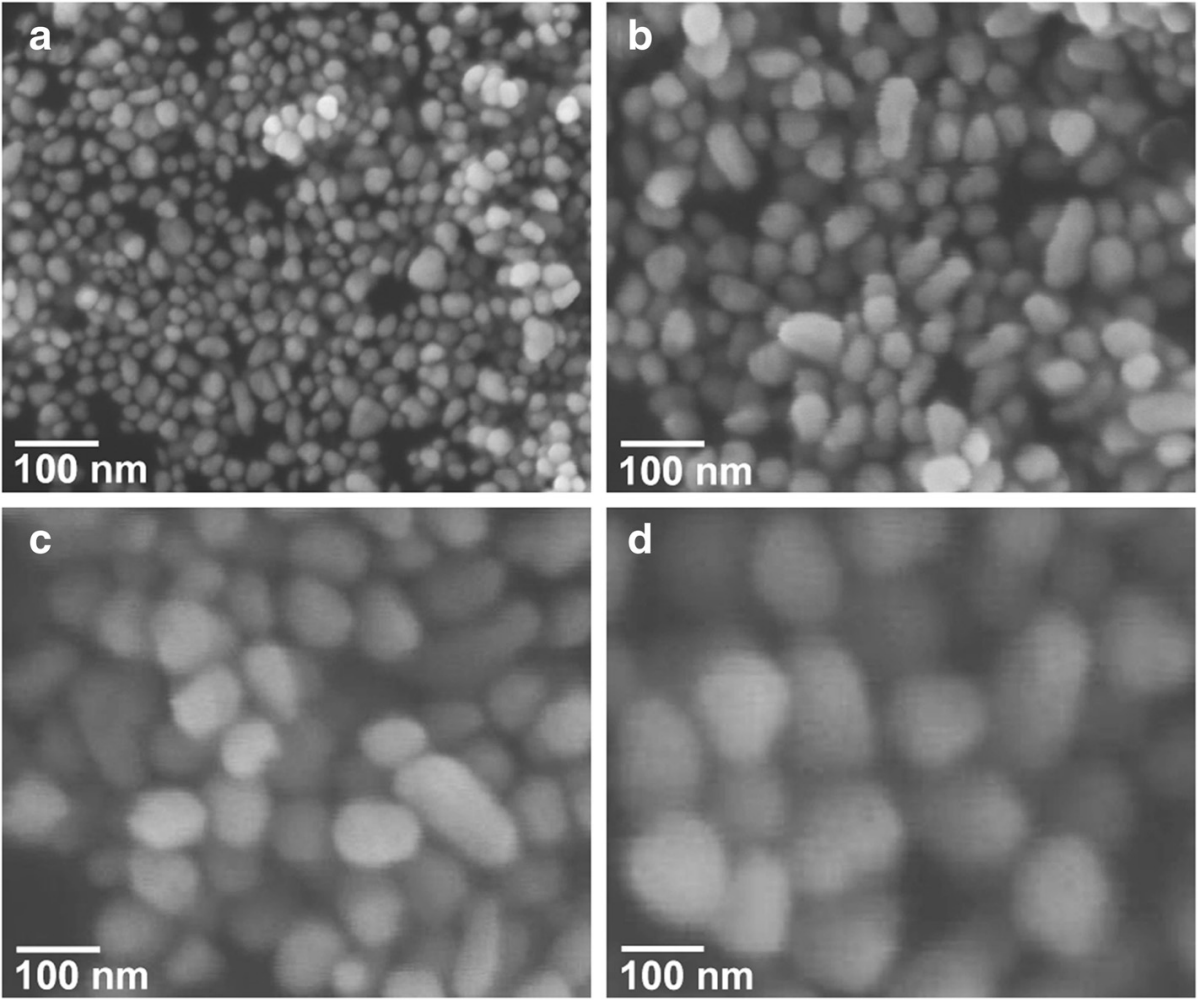
**SEM images.** Ni nanoparticles formed at **(a)** 240°C, **(b)** 255°C, **(c)** 270°C, and **(d)** 285°C.

**Figure 3:**
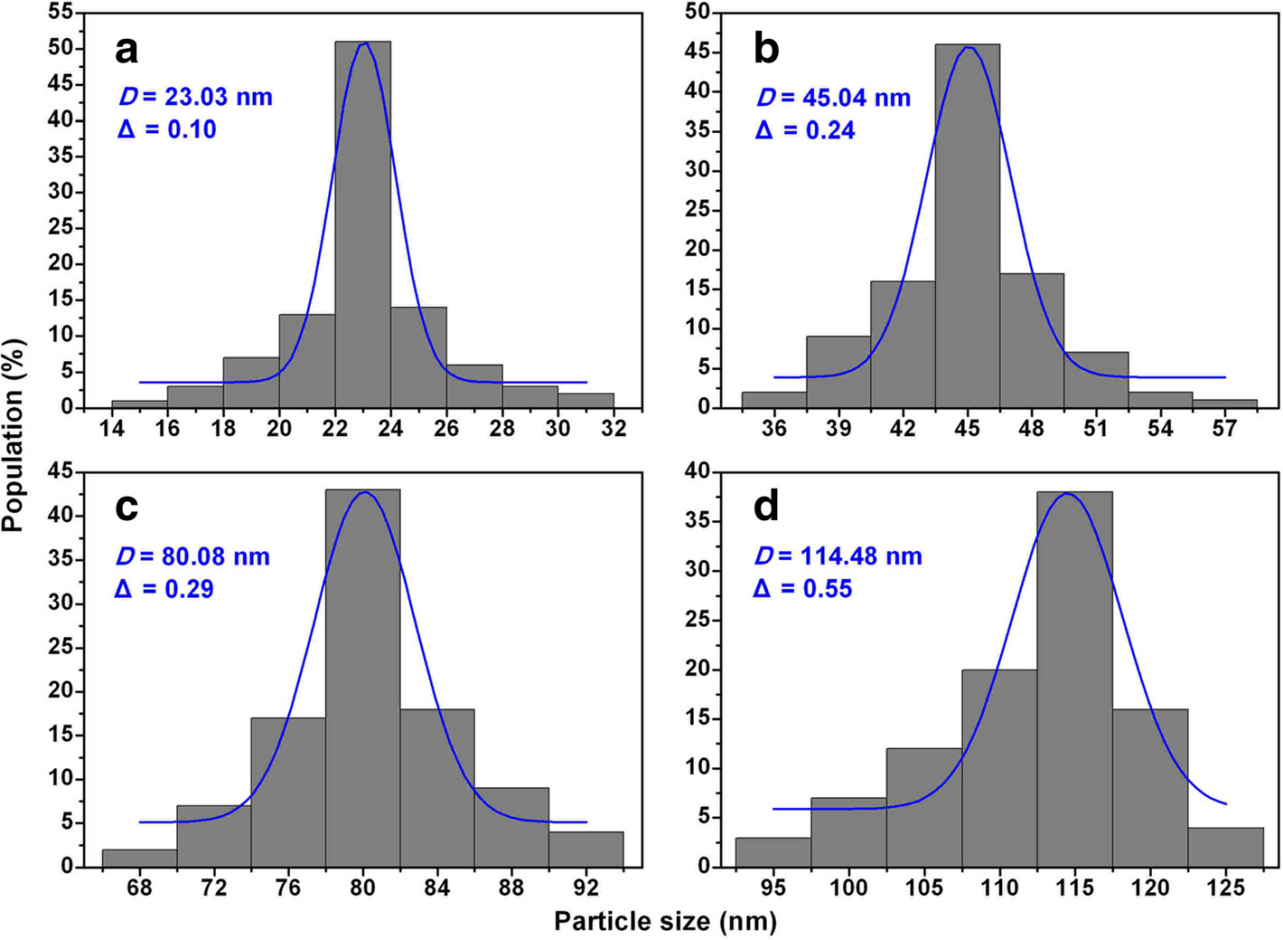
**Size distribution histograms.** Ni nanoparticles formed at **(a)** 240°C, **(b)** 255°C, **(c)** 270°C, and **(d)** 285°C. The blue lines represent the results of Gaussian fitting.

Figure 2 shows the representative SEM images of the Ni nanoparticles formed at 240°C, 255°C, 270°C, and 285°C. It is observed that most Ni particles are oval or spherical in shape. The particle-size analysis based on SEM images shows normal Gaussian distributions (Figure 3). It is apparent that the mean particle size increases with increasing reaction temperature, in excellent agreement with the results of XRD analysis. Hereinafter, *D* denotes the mean particle sizes which are 23, 45, 80, and 114 nm for the samples formed at 240°C, 255°C, 270°C, and 285°C, respectively, and the symbol ‘Δ’ denotes the absolute deviation obtained from the Gaussian fitting. It can be seen that the absolute deviation increases with the increase of mean particle size. It is reasonable to deduce that the change of reaction temperature will result in density difference of organic functional groups on the particle surface [31]. We propose that the control of reaction temperature is a mean for easy regulation of particle size and size distribution during the growth process.

**Figure 4:**
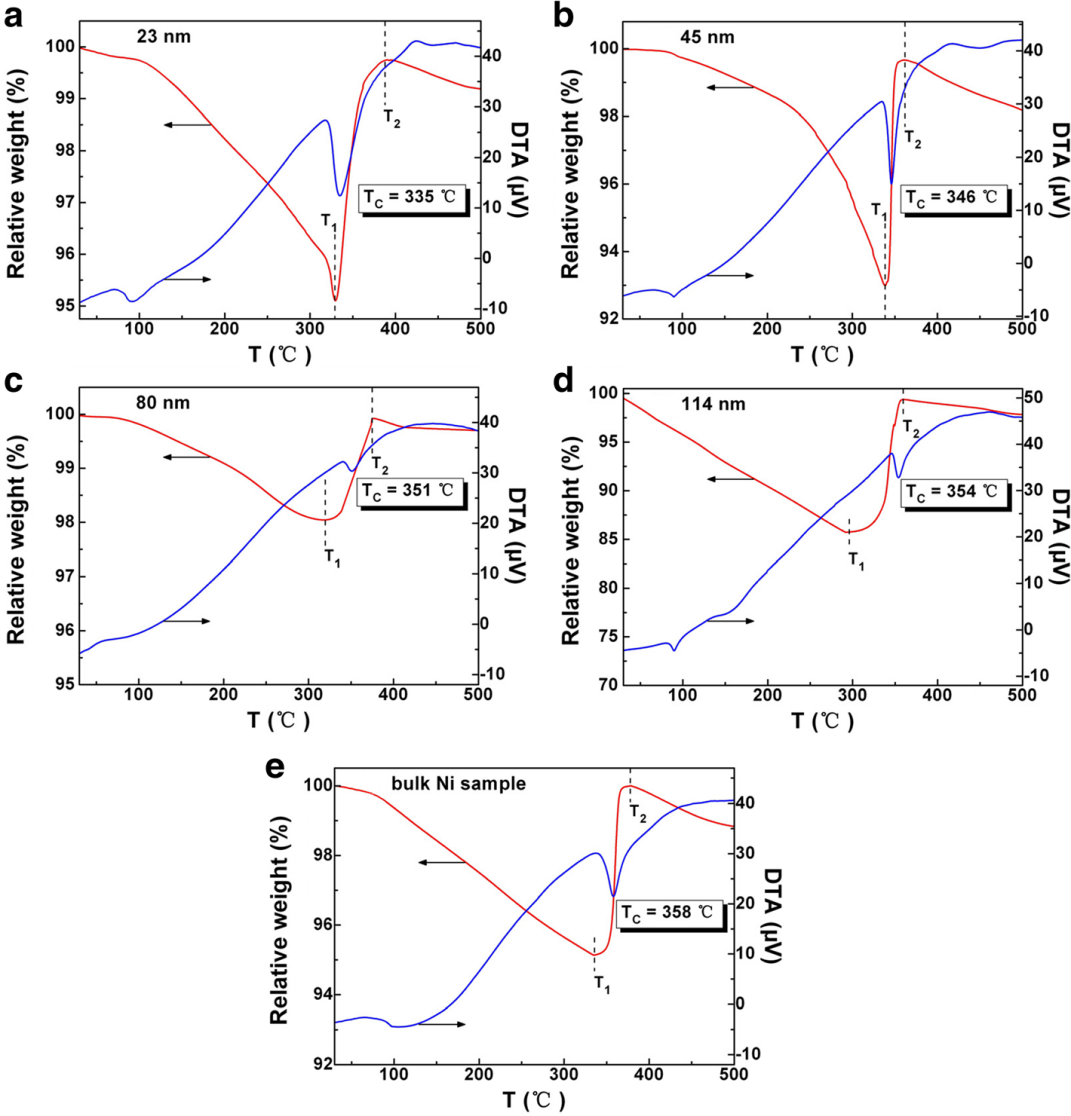
**TG/DTA curves.** Ni nanoparticles with particle sizes of **(a)** 23, **(b)** 45, **(c)** 80, **(d)** 114 nm, and **(e)** the corresponding bulk Ni sample, obtained in the presence of a piece of Nd_2_Fe_14_B permanent magnet and under argon atmosphere.

The TG/DTA curves were obtained with the samples kept under an argon atmosphere. In order to obtain information of Curie temperature, a piece of Nd_2_Fe_14_B permanent magnet was placed on top of the furnace chamber during the test. Usually, the magnetization per unit mass (or unit volume) decreases with increasing temperature. When the temperature reaches the Curie temperature of material, magnetization becomes zero and ferromagnetism disappears [32]. At that moment, there is no attraction between the magnet and the sample. As a result, the relative weight close to the Curie temperature will become larger. In the TG curves of Figure 4a,b,c,d, the relative weight decreases slowly at first and reaches a minimum at temperature *T*_1_, then increases quickly and reaches a maximum at temperature *T*_2_, and shows a gentle decline with further rise of temperature. The DTA curves in Figure 4a,b,c,d show a phase-transition peak between *T*_1_ and *T*_2_, and the phase-transition temperature is exactly the Curie temperature *T*_C_. For the Ni nanoparticles with particle sizes of 23, 45, 80, and 114 nm, the *T*_C_ values are 335°C, 346°C, 351°C, and 354°C, respectively. For comparison, the TG/DTA curve of a bulk Ni sample is shown in Figure 4e. Obviously, the *T*_C_ values of these Ni nanoparticles are lower than that of bulk nickel (358°C).

**Figure 5:**
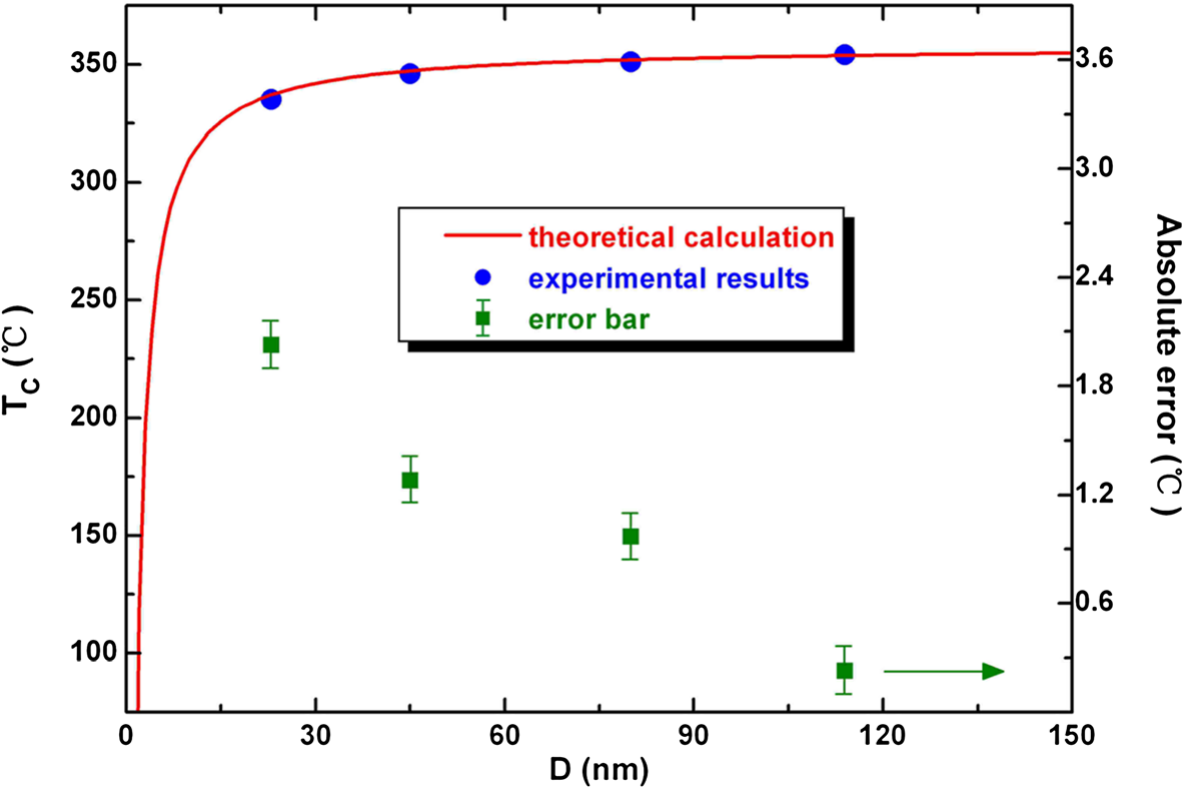
**Size dependence of Curie temperature.** Based on the TG/DTA experimental results (solid circles) and that of theoretical calculation (red solid line).

The *T*_C_ value decreases with the decrease of particle size (see the solid circles in Figure 5), showing a strong size effect. In theory, the cohesive energy *E*_n_ of free-standing nanoparticles with random shape can be described as [33]

(1) $$E_{n}=E_{b}\left(1-\frac{6\mu }{n^{1/3}C^{2/3}\pi k^{2}}\right),$$

where *E*_b_ denotes the cohesive energy of the corresponding bulk materials, *μ* is the shape factor, *n* is the atomic number of nanocrystals, *C* is the atomic number of the structure cell, and *k* is the ratio between equivalent atomic radius and lattice parameter. Considering that the Curie temperature is proportional to the cohesive energy [34], it is reasonable to express the Curie temperature *T*_Cn_ of nanoparticles with both size and shape dependence as

(2) $$T_{\mathrm{Cn}}=T_{\mathrm{Cb}}\left(1-\frac{6\mu }{n^{1/3}C^{2/3}\pi k^{2}}\right),$$

where *T*_Cb_ is the Curie temperature of the corresponding bulk materials. Further, for spherical nanoparticles, the atomic number *n* can be expressed as

(3) $$n=\frac{\rho \frac{4}{3}\pi {\left(\frac{D}{2}\right)}^{3}}{M}N_{A},$$

where *ρ* is the density of materials, *D* denotes the particle size of nanoparticles, *M* is the molar mass of matter, and *N*_*A*_ is the Avogadro constant. Considering our samples of spherical Ni nanoparticles with *fcc* structure, the *μ*, *C*, and *k* are 0.806, 4, and $\sqrt{2}/4$[35], respectively. Accordingly, substituting the correlative parameters (*ρ* = 8.908 g/cm^3^, *M* = 58.69 g/mol [36], *N*_*A*_ = 6.02 × 10^23^ mol^−1^, *T*_Cb_ = 358°C [25]) into Equations (2) and (3), the Curie temperature *T*_C_ of Ni nanoparticles with particle size *D* can be expressed as

(4) $$T_{C}=358-482.38/D.$$

The variation of *T*_C_ as a function of particle size calculated using Equation (4) is compared with the TG/DTA results. Specifically, the absolute error between the two results has been given by the error bars in Figure 5, and the obtained relative errors are less than 1%. Through this, we can understand that *T*_C_ decreases with decrease of particle size *D*, and there is a good match between theoretical and experimental results. In other words, the model represents well the size dependence of the Curie temperature of Ni nanoparticles.

**Figure 6:**
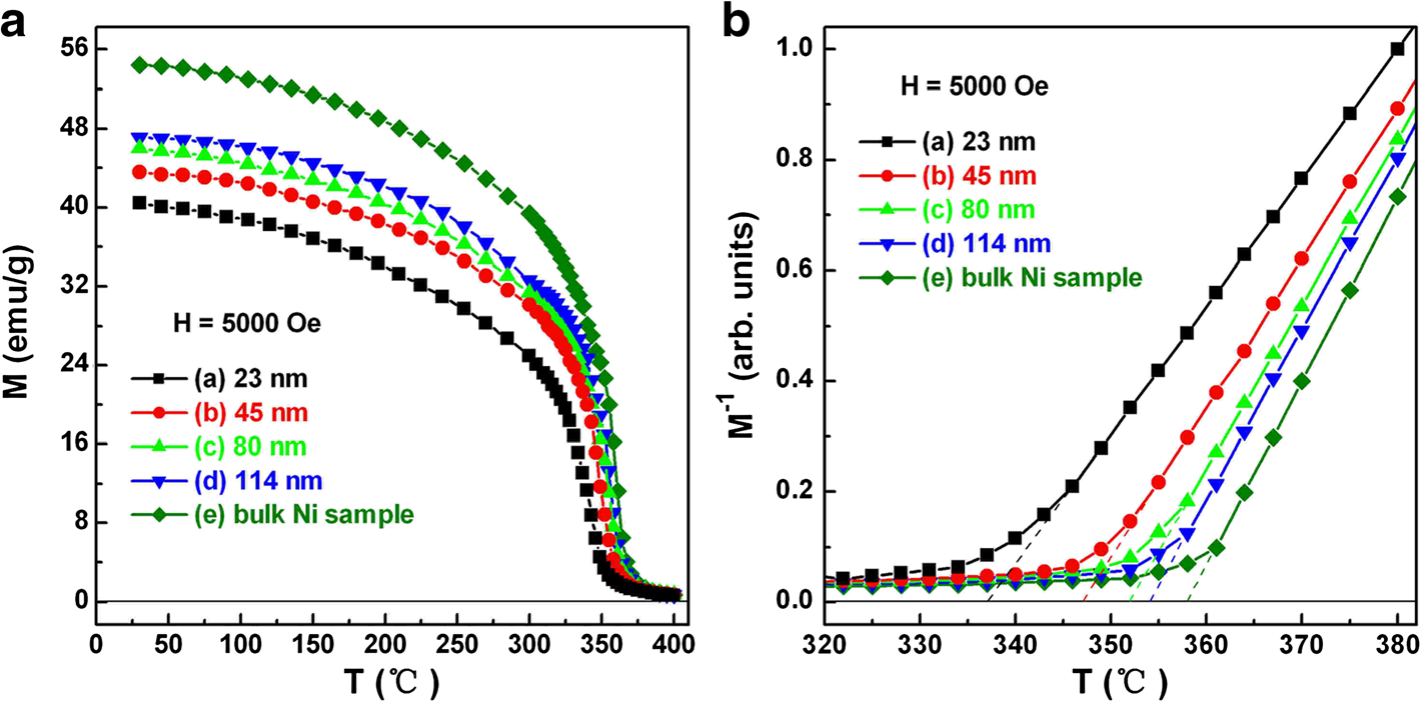
***M*-*T*(a) and*M***^**−1**^**-*T*(b) curves.** Ni nanoparticles with particle sizes of (a) 23, (b) 45, (c) 80, (d) 114 nm, and (e) the corresponding bulk Ni sample.

Further, the temperature-dependent magnetization was studied by VSM. Figure 6a shows the typical *M*-*T* curves of the Ni nanoparticles with various sizes and the corresponding bulk Ni sample. To get the accurate *T*_C_, the *M*^−1^-*T* curves are also given in Figure 6b. According to the Curie-Weiss law, the horizontal axis intercept of *M*^−1^-*T* curve is the Curie temperature *T*_C_ of ferromagnetic materials. Therefore, the *T*_C_ values are observed to be 337°C, 347°C, 352°C, and 354°C for the Ni nanoparticles with sizes of 23, 45, 80, and 114 nm, respectively. At the same time, the *T*_C_ of bulk Ni sample remains 358°C. It goes without saying that the variation trend and the absolute value of *T*_C_ confirm the results of TG/DTA measurements.

**Figure 7:**
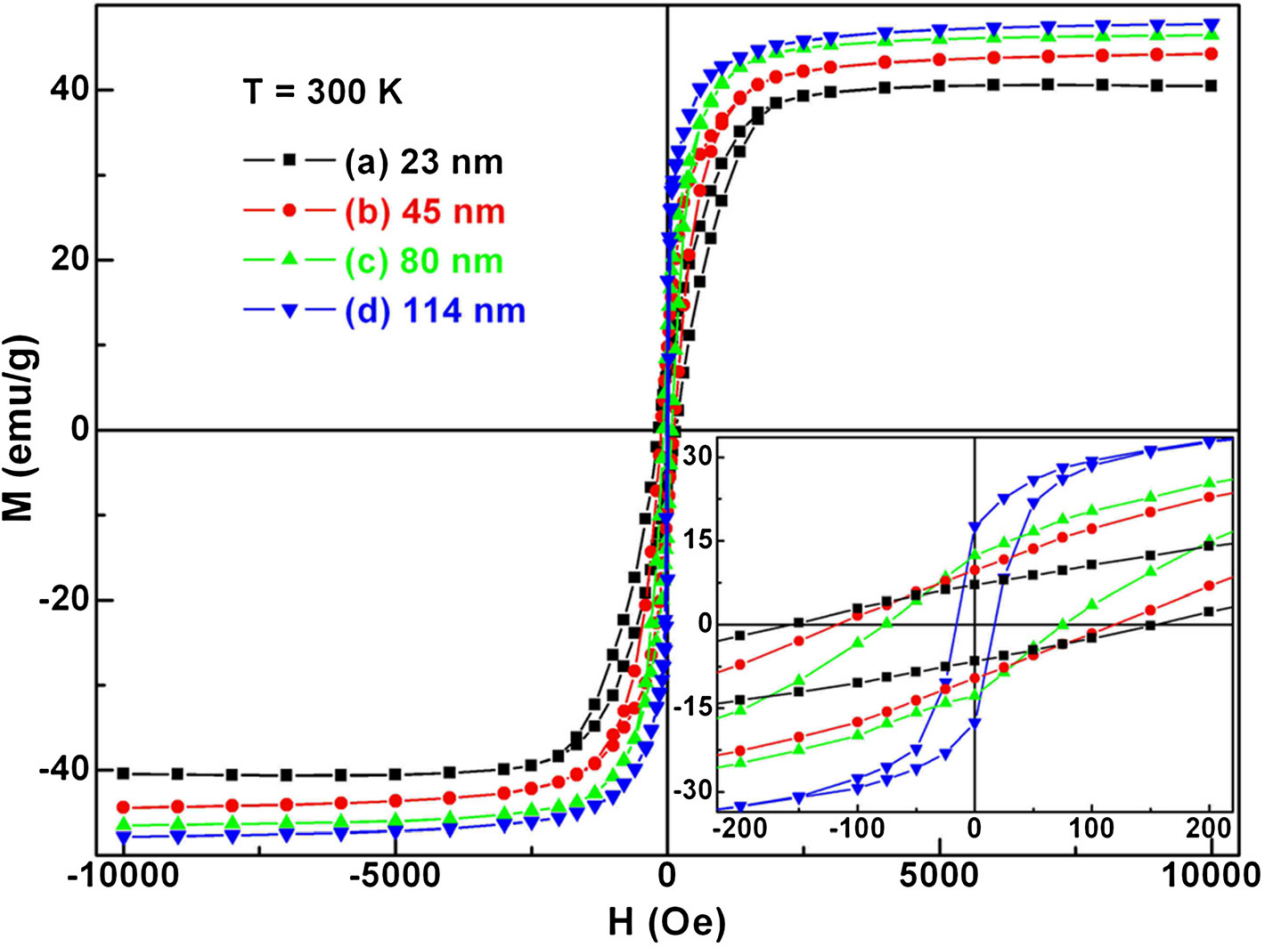
**Room-temperature hysteresis loops.** Ni nanoparticles with particle sizes of (a) 23, (b) 45, (c) 80, and (d) 114 nm. Inset shows greater detail of the measurements around the origin.

Nickel is an important magnetic material. To further investigate the magnetic properties of Ni nanoparticles, magnetic measurements of the four Ni-nanoparticle samples with different particle sizes were carried out. Figure 7 shows their room-temperature hysteresis loops. All four samples show hysteresis behavior, revealing that the nanoparticles are ferromagnetic. With increasing particle sizes, the magnetization of the samples increases with applied field. The saturation magnetization *M*_S_ for all samples are listed in Table 1. The *M*_S_ value of sample *d* determined at room temperature in a field of 1 T is close to that of bulk nickel (*M*_Sb_ = 54.4 emu/g) [8], indicating the metallic characteristic at this state of Ni nanoparticles. The relatively lower *M*_S_ of 40.47 emu/g for sample *a* can be reasonably considered to be due to the smaller particle size as well as to the accompanied increase of specific surface area. The decrease of *M*_S_ with decreasing particle size is consistent with that reported previously [37].

**Figure 8:**
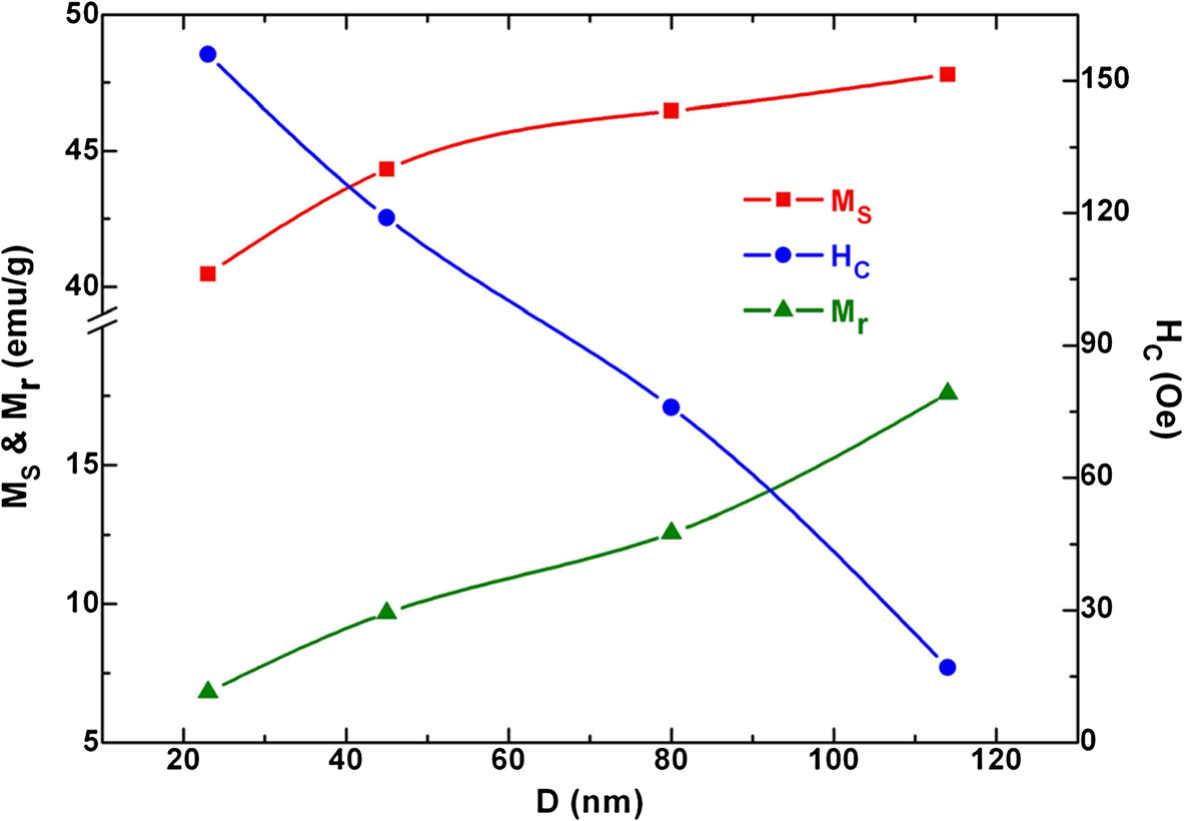
**Size dependence of magnetic parameters.** Saturation magnetization *M*_S_, remanent magnetization *M*_r_, and coercivity *H*_C_ in the case of spherical Ni nanoparticles.

Based on the inset of Figure 7, the remanent magnetization *M*_r_ and coercivity *H*_C_ of all Ni-nanoparticle samples are obtained and also listed in Table 1. As shown in Figure 8, the *M*_S_, *M*_r_, and *H*_C_ of spherical Ni nanoparticles are size-dependent. More specifically, the *M*_S_ and *M*_r_ increase and the *H*_C_ decreases monotonously with increasing *D*, indicating a distinct size effect. According to the effect of particle size on the magnetic coercivity [38], the *H*_C_ of the multidomain ferromagnetic nanoparticles conforms to the rule of *H*_C_ ∝ 1/*D*. However, the single domain size of nickel, 21.2 nm, has been calculated by the magnetic domain theory [37]. Obviously, the particle size for the all current Ni samples is larger than this value. Hence, it is understandable to observe a decline of coercivity with rise of particle size in the case of Ni nanoparticles.

In order to rationalize the decrease of saturation magnetization with decreasing particle size in proportion to the specific surface area of the particles, a magnetically dead layer theory has been developed [39]. In the theory, the saturation magnetization *M*_Sn_ of nanoparticles follows the formula [40]

(5) $$M_{\mathrm{Sn}}=M_{\mathrm{Sb}}\left(1-6t/D\right),$$

where *M*_Sb_ denotes the saturation magnetization of the corresponding bulk materials, *t* is the thickness of magnetically inactive layer, and *D* is the diameter of nanoparticles. Considering our samples of spherical Ni nanoparticles (*M*_Sb_ = 54.4 emu/g) [8], the percentage of magnetically inactive layer *t*/*D* can be calculated using Equation (5). The *t*/*D* values for all samples are listed in Table 1. Clearly, as the surface-to-volume ratio of Ni nanoparticles increases with decreasing particle size, the percentage of magnetically inactive layer increases too. In fact, as the particle size reduced from 114 to 23 nm, the *t*/*D* value of Ni nanoparticles slightly increases from 2.03% to 4.26%. This slight change suggests that the dead layer theory cannot satisfactorily explain the size dependence of saturation magnetization [39-41].

Previous studies revealed that the size-dependent effect of saturation magnetization is attributable to the decrease of cohesive energy [42,43]. Generally, the size-dependent cohesive energy *E*_n_ of spherical nanoparticles can be described as

(6) $$\frac{E_{n}}{E_{b}}=\left(1-\frac{1}{2D/h-1}\right)\exp\left(-\frac{2S_{\mathrm{vib}}}{3R}\frac{1}{2D/h-1}\right),$$

where *S*_vib_ denotes the vibrational part of the overall melting entropy *S*_m_, *R* is the ideal gas constant, and *h* denotes the atomic diameter. By incorporating the bond order-length-strength (BOLS) correlation mechanism into the Ising convention and the Brillouin function [44,45], a simplified model can be developed to describe the relationship between the saturation magnetization *M*_Sn_ of spherical nanoparticles and the average size *D* of nanoparticles:

(7) $$\frac{M_{\mathrm{Sn}}}{M_{\mathrm{Sb}}}=4\left(1-\frac{1}{2D/h-1}\right)\exp\left(-\frac{2S_{\mathrm{vib}}}{3R}\frac{1}{2D/h-1}\right)-3.$$

Considering our samples of ferromagnetic Ni nanoparticles, the relevant parameters are *M*_Sb_ = 54.4 emu/g [8], *S*_vib_ ≈ *S*_m_ = 10.12 J mol^−1^ K^−1^[46], and *h* = 0.2492 nm [36]. Substituting *R* = 8.314 J mol^−1^ K^−1^ into Equation (7), the *M*_S_ of Ni nanoparticles with size *D* can be expressed as

(8) $$M_{S}=217.6\left(1-\frac{1}{8.0257D-1}\right)\exp\left(\frac{0.8115}{1-8.0257D}\right)-163.2.$$

**Figure 9:**
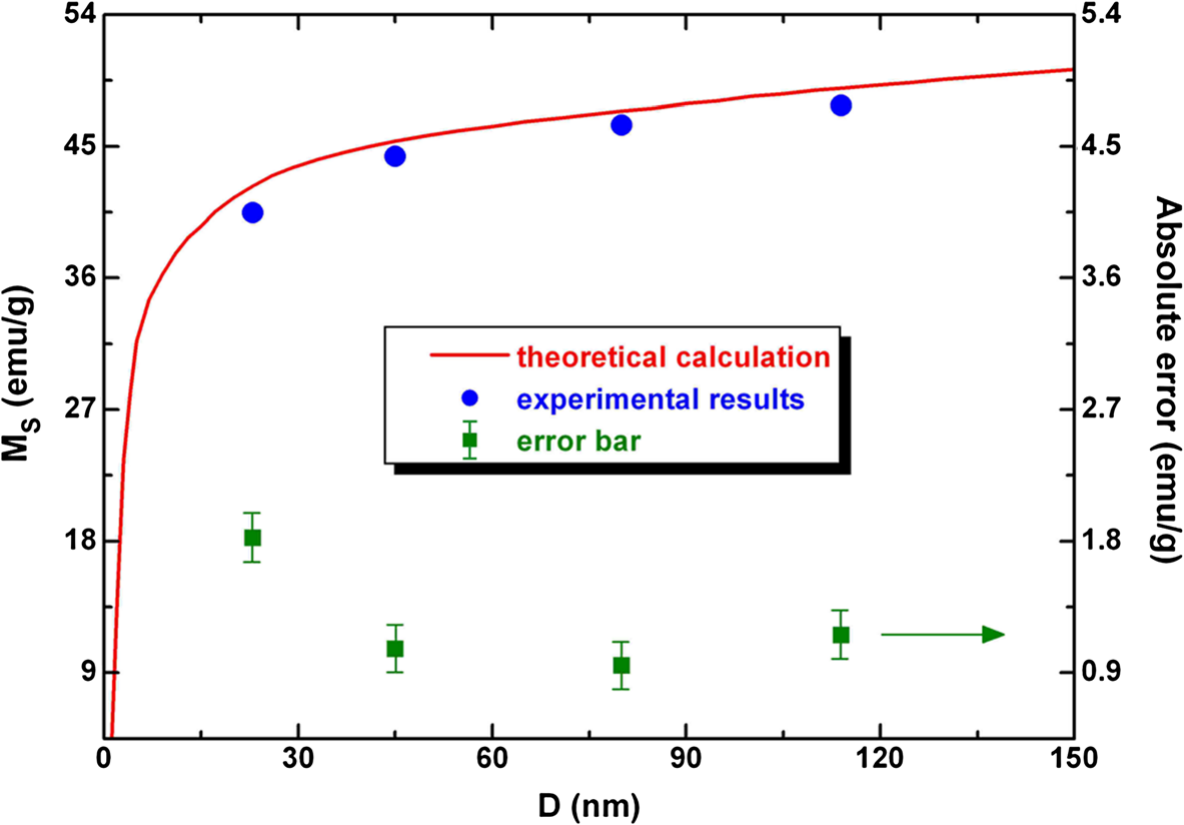
**Size dependence of saturation magnetization.** For the VSM experimental results (solid circles) and that of theoretical calculation (red solid line).

Shown in Figure 9 is a comparison between the theoretical results based on Equation (8) and the results of VSM measurement. The error bar reveals the difference between the two results, and the maximum value of relative error is 4.52%. Thus, it can be seen that the model prediction is in agreement with the experimental results. In other words, the size-dependent cohesive energy model can describe reasonably well the size dependence of Ni nanoparticles in terms of saturation magnetization. The agreement between the theoretical calculation and experimental results suggests that the drop of *M*_S_ is essentially induced by the increase of surface-volume ratio, the same as the size dependence of any thermodynamic amount [47].

Based on the mathematical relation of exp(−*x*) ≈ 1 − *x*_,_ when *x* is small enough as a first-order approximation, Equation (7) can be rewritten as

(9) $$\frac{M_{\mathrm{Sn}}}{M_{\mathrm{Sb}}}\approx 1-\frac{2h}{D}\left(1+\frac{2S_{\mathrm{vib}}}{3R}\right).$$

Nonetheless, it should be noted that the difference between Equations (7) and (9) becomes evident when the size of nanoparticles further decreases to about several nanometers or smaller scale. Let Equation (9) be equal to zero, namely *M*_Sn_(*D*_c_) = 0, where *D*_c_ denotes the critical size,

(10) $$D_{c}\approx 2h\left[1+2S_{\mathrm{vib}}/\left(3R\right)\right].$$

Substituting the parameters mentioned above into Equation (10), the *D*_c_ value of spherical Ni nanoparticles is 0.90 nm. As shown in the final column of Table 1, the percentage of magnetically inactive layer is 4.26%, 3.09%, 2.43%, and 2.03% for spherical Ni nanoparticles with particle sizes of 23, 45, 80, and 114 nm, respectively. Using Equation (5), the corresponding thickness *t* of magnetically inactive layer is 0.98, 1.39, 1.94, and 2.31 nm for the four Ni-nanoparticle samples. Herein, the critical size (*D*_c_ = 0.90 nm) and *t* values are in the same order of magnitude, but the *D*_c_ value is significantly smaller than the four *t* values. Thus, the size-dependent cohesive energy model under critical condition is consistent with the magnetically dead layer theory.

## Conclusions

We systematically studied the size-dependent magnetic properties of spherical Ni nanoparticles in terms of experimental measurement and theoretical calculation. Our results show that the Curie temperature, saturation magnetization, and remanent magnetization increase whereas the coercivity decreases monotonously with the increase of particle size. According to the size-dependent cohesive energy model, a simplified theoretical calculation can be applied to analyze the size dependence of Curie temperature and saturation magnetization. The results of calculation are in good agreement with the experimental results. Under critical condition of critical size *D*_c_ = 0.90 nm, the size dependence of magnetization obtained by cohesive energy model is consistent with the analysis of magnetically dead layer theory. With the particle size decreasing, the surface-to-volume ratio of Ni nanoparticles increases and the percentage of magnetically inactive layer increases as well.

## Competing interests

The authors declare that they have no competing interests.

## Authors' contributions

XH carried out the experiment and prepared the manuscript. WZ participated in the design of the study and helped to draft the manuscript. CA and YD helped in the discussion and analysis of the experimental results. All authors read and approved the final manuscript.
